# Polymorphisms *FTO* rs9939609, *PPARG* rs1801282 and *ADIPOQ* rs4632532 and rs182052 but not lifestyle are associated with obesity related-traits in Mexican children

**DOI:** 10.1590/1678-4685-GMB-2015-0267

**Published:** 2016-07-14

**Authors:** C Muñoz-Yáñez, R Pérez-Morales, H Moreno-Macías, E Calleros-Rincón, G Ballesteros, R. A González, J Espinosa

**Affiliations:** 1 Departamento de Investigación, Facultad de Medicina, Universidad Juárez del Estado de Durango, Durango, México; 2 Departamento de Biología Molecular, Facultad de Ciencias Químicas, Universidad Juárez del Estado de Durango, Durango, México; 3 Departamento de Economía, División CSH de la Universidad Autónoma Metropolitana, Unidad Iztapalapa, D.F. México, México; 4 Facultad de Ciencias, Universidad Autónoma del Estado de Morelos, Cuernavaca, Morelos, México

**Keywords:** Obesity, children, polymorphisms, energy intake, physical activity, lipid profile

## Abstract

Concerning the genetic factors of obesity, no consistent association between populations has been reported, which may be due to the frequency of polymorphisms, the lifestyle of studied populations and its interaction with other factors. We studied a possible association of polymorphisms *FTO* rs9939609, *PPARG* rs1801282, and *ADIPOQ* rs4632532 and rs182052 with obesity phenotypes in 215 Mexican children. Glucose, triglycerides, cholesterol, HDL and LDL were measured. In addition, weight, height, waist circumference and triceps skin thickness were recorded. High-energy diets and sedentary behavior were evaluated with a validated questionnaire. In contrast with other reports, only *FTO* rs9939609 was associated with obesity related-traits, including BMI (*p* = 0.03), waist circumference (*p* = 0.02), triceps skinfold (*p* = 0.03) and waist/height ratio (*p* = 0.01), and also with cholesterol levels (*p* = 0.02) and LDL (*p* = 0.009). Lower levels of triglycerides (p=0.04) were related with presence of *PPARG* rs1801282, while *ADIPOQ* rs4632532 showed an effect on HDL (*p* = 0.03) levels. On the other hand, diet, physical activity and screen time were not related with obesity. In summary, only *FTO* rs9939609 was associated with obesity related-traits, while *PPARG2* rs1801282 and *ADIPOQ* rs4632532 were involved in lipid metabolism.

## Introduction

Obesity is a worldwide public health problem due to its association with chronic diseases, such as type 2 diabetes, hypertension, cardiovascular disorders and certain types of cancer. Obesity results from an interaction of social, psychological, genetic and environmental factors, like diet and physical exercise. The last National Health and Nutrition Survey found that the national prevalence of childhood overweight did not increase. However, in the Mexican state of Durango the childhood obesity raised from 22.9% in 2006 (Shamah-Levy and Villalpando-Hernández, 2007) to 37.5% in 2012 (Rivero-Vázquez, 2013), which might be the result of different genetic backgrounds of Mexico's Northern populations (Silva-Zolezzi *et al.*, 2009). Many studies have demonstrated that genetic factors have an important role in the development of obesity, although results are different depending on the population being evaluated.

One of the most studied genes, the *PPARG* is a member of the nuclear hormone receptors super-family, which regulates transcription of genes involved in several biological functions, such as cell growth, adipocyte differentiation, metabolism of cholesterol and fatty acids, cell survival, ubiquitination and adaptive thermogenesis. *PPARG* is activated by lipophilic hormones, fatty acids from the diet, and their metabolites (He, 2009). The Ala allele of the polymorphism Pro12Ala of *PPARG* was associated with obesity in a population from Spain (OR = 2.36, p = 0.03) and from India (OR = 3.2, p = 0.02) (González-Sánchez *et al.*, 2002; Bhatt *et al.*, 2012), while the Pro allele was a risk factor for type 2 diabetes in a French population (OR = 1.37, p = 0.04) (Ghoussaini *et al.*, 2005).

*FTO* was the first gene to be associated with obesity by genome-wide association studies (GWAs). Adults homozygous the A allele, in rs9939609 polymorphism, weight on average 3 – 4 kg more and have a 1.67-fold increased risk of obesity (Frayling *et al.*, 2009). However, *FTO* functions are not yet fully described; an *in vitro* study showed that it acts as a co-activator of the C/EBP family of transcriptional regulators, which in conjunction with *PPARG* are necessary for adipocyte differentiation (Wu *et al.*, 2010). In addition, it is expressed in proopiomelocortin-producing neurons participating in the satiety cycle (Tung *et al.*, 2010). Accordingly, epidemiological studies support an association of the A allele with increased caloric intake (Speakman *et al.*, 2008), and loss of overeating control (Tanofsky-Kraff *et al.*, 2009). *FTO* rs9939609 was also associated with risk of obesity in a Mexican (OR = 1.38, p = 0.03) and Chinese populations (OR = 1.29, p = 0.001) (Villalobos-Comparán *et al.*, 2008; Xi *et al.*, 2010)

The less studied *ADIPOQ* gene encodes adiponectin, a hormone secreted by adipose tissue into plasma, where it binds with receptors in muscle and liver, and participates in glucose uptake and beta-oxidation of lipids. Adiponectin levels were associated with obesity and insulin resistance in an Italian population (Filippi *et al.*, 2004). In a Mexican American population, *ADIPOQ* polymorphisms rs4632532 and rs182052 increased BMI (p = 0.032 and p = 0.029, respectively) (Richardson *et al.*, 2006);. Polymorphism rs182052 was related with low expression levels of adiponectin, in a Chinese population (Ong *et al.*, 2010).

In this study, we investigated the association between polymorphisms *FTO* rs9939609, *PPARG2* rs1801282, and *ADIPOQ* rs4632532 and rs182052 with obesity-related traits in a Mexican population with high prevalence of childhood obesity.

## Materials and Methods

### Subjects

Two hundred and fifteen Mexican mestizo children (108 males and 107 females, ranging from 6.1 to 12.3 years old), whose parents and grandparents were born in Mexico, were selected to participate in the study. Sampling was performed on ten public elementary schools from the bordering cities of Gómez Palacio and Lerdo, in the state of Durango, Mexico, between January and June 2012. The protocol was approved by the Ethics Committee of the Facultad de Medicina of the Universidad Autónoma de Coahuila, Mexico, and all parents signed an informed consent.

The BMI is an attempt to quantify the amount of tissue mass (muscle, fat, and bone) in an individual. It is defined as the body mass divided by the square of the body height, and is universally expressed in units of kg/m^2^. The waist/height ratio is a better predictor of a person's metabolic risk (Browning *et al.*, 2010) and is defined as waist circumference (cm) divided by height (cm). Triceps skinfold thickness is measured in the right arm with Lange calipers. All children were assessed and measurements were recorded according to an established protocol (Shamah-Levy and Villalpando-Hernández, 2007). The "overweight/obese" group was established using the age- and sex-specific BMI cutoff specified by the International Obesity Task Force (IOTF) (Cole *et al.*, 2000) and by the World Health Organization (De Onis *et al.*, 2007). Blood samples were drawn after overnight fasting. Total cholesterol, HDL (both by CHOD-PAP, Randox Laboratories Ltd. Ardmore, Crumlin, UK) and Triglyceride (Pointe Scientific Inc. Detroit, Michigan, US) levels were evaluated by enzymatic colorimetric methods. LDL levels were calculated using Friedewald's formula. Glucose levels were measured by Glucose Oxidase method (Randox Laboratories Ltd. Ardmore, Crumlin, UK). Plasma glucose levels were measured within 2 hours after the sample was obtained and plasma samples were stored at −20°C until analysis for the other biochemical parameters.

Dietary information was obtained by a semi-quantitative food frequency questionnaire (FFQ) applied to mothers or caregivers by trained personnel. The questionnaire included 101 food items classified in 14 groups and was used by the National Institute of Public Health in The National Survey of Health and Nutrition 2006 (ENSANUT2006) (Shamah-Levy and Villalpando-Hernández, 2007). The interviewers asked about portions that were consumed by the children (times per week, times per day, and size) for each food item during the seven-day period prior to the interview. Dietary energy intake and source were calculated using the free specialized SNUT software (Rivera-Dommarco *et al.*, 2001). The number of hours per week that children engaged in intense physical activity were also asked, and recorded as Metabolic Equivalent (MET)/hour (a unit of MET represents a multiple of the oxygen consumption when at rest, which corresponds to 3.5 mL O_2_kg^−1^min^−1^. For example, if a person exercising expends 10 METs, he /she is using 10 times the amount of oxygen consumed when at rest) (Ridley *et al.*, 2008; Morales-Ruán *et al.*, 2009). Energy intake was assessed considering plausible intake levels according to the methodology used for ENSANUT2006 (Rodríguez-Ramírez *et al.*, 2009). Children with reported energy intake above 5 standard deviations (outliers) or below 25% of the recommended caloric intake (extreme malnutrition) were excluded from the obesogenic environment analysis because these values are not biologically plausible (n=164). Atypical food consumptions were manually reviewed and when a clear mistake was detected or if the value was not plausible, the participant was excluded.

### Genotyping assays

Genomic DNA was isolated from peripheral white blood cells (3mL blood in EDTA) using the salting out method (Lahiri and Nurnberger, 1991). Genotyping assays were performed with TaqMan probes in the ABI Prism 7900HT sequence Detection System (Applied Biosystems, Foster City, CA, USA) using the probes C_1129864_10 for *PPARG* rs1801282, C_30090620_10 for *FTO* rs9939609, C_2412785_10 for *ADIPOQ* rs4632532 and C_27807233_10 for *ADIPOQ* rs182052 according to manufacturer's instructions (Applied Biosystems).

### Statistical analysis

The quantitative variables were reported as medians and interquartile ranges. Allele and genotype frequencies were estimated by direct gene counting. The Hardy-Weinberg equilibrium was estimated using a Chi-square test. All the analyses were performed considering the cutoffs for obesity established by IOTF. Logistic regression models adjusted for age and gender were used to test associations of each genotype with overweight/obesity. We also analyzed the effect of polymorphisms on several anthropometric (waist circumference, arm circumference, triceps skinfold thickness, waist circumference/height ratio) and metabolic parameters (glucose, triglycerides, cholesterol, HDL, LDL) using linear regressions under an additive model adjusted by gender and age. All quantitative traits were logarithmically transformed before statistical analysis because some variables did not follow a normal distribution. Association analyses were performed using the STATA 11.1 software. Statistical significance was considered at *p*-values < 0.05 for all comparisons.

## Results

The prevalence of overweight/obesity in the 215 evaluated children was 43.72% and 39.07% based in the IOTF and WHO cutoffs, respectively (Table 1); no significant difference was observed in the association analysis using the different cutoffs. The obesogenic environment analysis showed that children consumed more fat and less protein than recommended (Casanueva and Kaufer, 2008),. Also, screen time was above the limit established by the American Association of Pediatrics (2h/day), and triceps skinfold thickness was higher than the normal for the median age (Casanueva *et al.*, 2008). No significant associations were observed between obesogenic environment and obesity or related traits (data not shown).

**Table 1:** General characteristics of the population studied.

Variables	n (%)	
**Gender**		
	Male	108 (50.23)
	Female	107 (49.77)
**IOTF cutoff**		
	Normal weight	121 (56.28)
	Overweight/Obesity	94 (43.72)
**WHO cutoff**		
	Normal weight	131 (60.93)
	Overweight/Obesity	84 (39.07)
	**Median (Min-Max)**	
**Age (years)**	10.4 (6.10–12.30)	
**Anthropometric**		
Waist circumference (cm)	64.25 (48.95–96.85)	
Arm circumference (cm)	21.95 (13.50–35.50)	
Triceps (mm)	15.00 (5.50–28.00)	
Waist/height ratio	0.46 (0.35–0.63)	
**Obesogenic Environment** *		
Energy intake (kJ/d)	8837.62 (4235.32–19608.62)	
Carbohydrates (%)	47.14 (21.37–73.14)	
Protein (%)	13.81 (8.48–20.67)	
Fat (%)	43.05 (20.18–64.52)	
Physical activity (h/week)	5 (1–6)	
Screen hours (h/week)	17 (2–32)	

* n=164 children included. IOTF: International Obesity Task Force; WHO: World Health Organization.

Regarding the genetic background, we found a very high percentage of children with familiar history of obesity-related diseases such as diabetes mellitus (63.96%) and hypertension (59.90%). Some children had levels of biochemical parameters above the reference values (International Diabetes Federation) (Zimmet *et al.*, 2007) (Table 2). The minor allele frequencies were 0.22 for *PPARG rs1801282*, 0.35 for *FTO* rs9939609, 0.61 for *ADIPOQ* rs4632532 and 0.63 for *ADIPOQ* rs182052, which are within range from those reported in other populations (Table 3). Genotype frequencies in all genes were in accordance with the Hardy-Weinberg equilibrium.

**Table 2:** Biochemical parameters and hereditary backgrounds.

Biochemical parameters	Median (Min-Max)	% Above reference value*
Glucose (mg/dL)	79.87 (58.34–113.07)	5 (2.33)
Cholesterol (mg/dL)	148.49 (84.80–353.70)	25 (11.63)
Triglycerides (mg/dL)	78.29 (26.58–221.60)	16 (7.44)
HDL (mg/dL)	53.66 (23.95–104.75)	46 (21.40)
LDL (mg/dL)	74.21 (24.38–299.34)	14 (6.51)
**Familial history**	**n (%)**	
Diabetes mellitus	126 (63.96)	
Hypertension	118 (59.90)	
Dyslipidemia	73 (37.06)	
Cardiovascular	42 (21.32)	

* According to International Diabetes Federation (IDF)(Zimmet *et al.*, 2007)

**Table 3:** Comparison of association of *FTO* rs9939609, *PPARG2* rs1801282, and *ADIPOQ* rs4632532 and rs182052 with obesity in different populations.

Polymorphism	Allelic Frequency	Model	OR	IC 95%	Population	Reference
***PPARG* rs1801282**						
	G = 0.09	Additive	2.36	1.10–5.05	Caucasian	González-Sánchez, *et al.*, 2002
	G = 0.06	Dominant	2.85	1.07–7.62	Caucasian	Morini, *et al.*, 2008
	G = 0.05	Dominant	0.64	0.42–0.76	Chinese	Wang, *et al.*, 2013
	G = 0.11	Recessive	3.2	1.2–12.9	Indian	Bhatt, *et al.*, 2012
	**G = 0.22**	**Additive**	**1.25**	**0.73–2.15**	**Mexican – Mestizo**	**This study**
***FTO* rs9939609**	A = 0.19	Additive	1.41	1.15–1.76	Mexican – Mestizo	León-Mimila, *et al.*, 2013
	A = 0.20	Additive	2.42	1.71–3.44	Mexican – Mestizo	Villalobos-Comparán, *et al*., 2008
	A = 0.12	Additive	1.29	1.11–1.49	Chinese	Xi, *et al.*, 2010
	A = 0.42	Additive	1.27	1.20–1.34	Caucasian	Andreasen, *et al.,* 2008
	A = 0.59	Recessive	1.97	1.29–3.00	Caucasian	Luczynski, *et al.*, 2012
	**A = 0.35**	**Additive**	**1.27**	**0.82–1.98**	**Mexican – Mestizo**	**This study**
***ADIPOQ***						
rs4632532	T = 0.48	Codominant	Increase in BMI		Mexican – American	Richardson, *et al.*, 2005
	T = 0.45	Codominant	Increase in BMI		Hispano – American	Sutton, *et al.*, 2005
	**T = 0.61**	**Additive**	**1.47**	**0.78–2.74**	**Mexican – Mestizo**	**This study**
rs182052	G = 0.37	Additive	1.22	1.05–1.42	Afroamerican	Bostrom, *et al.*, 2008
	G = 0.48	Codominant	Increase in BMI		Mexican – American	Richardson, *et al.*, 2005
	G = 0.44	Codominant	Increase in BMI		Hispano – American	Sutton, *et al*., 2005
	**G = 0.63**	**Additive**	**1.43**	**0.76–2.74**	**Mexican – Mestizo**	**This study**

In respect to the association of genotypes with obesity, under an additive model, the OR was 1.25 (CI = 0.73 – 2.15) for *PPARG* rs1801282, 1.27 (CI = 0.82 – 1.98) for *FTO* rs9939609, 1.47 (CI = 0.78 – 2.74) for *ADIPOQ* rs4632532 and 1.43 (CI = 0.76 – 2.74) for *ADIPOQ* rs182052. A risk tendency was observed, although these values were not significant (Table 3). The association, however, was assessed under the codominant, dominant and recessive models and the risk trend was preserved (data not shown). The analysis of genotypes with obesity-related characteristics found that *FTO* was associated with BMI (β = 0.46, p = 0.03), waist circumference (β = 0.03, p = 0.02), triceps skinfold (β = 0.08, p = 0.03) and waist/height ratio (β = 0.03, p = 0.01) (Table 4). Additionally, *FTO* had an effect on total cholesterol (β = 0.06, p = 0.02) and LDL (β = 0.12, p = 0.01) levels, although the increase was modest; in contrast *ADIPOQ* rs4632532 and *PPARG* rs1801282 were associated with a slight decrease in levels of HDL (β = −0.06, p = 0.03) and triglycerides (β = −0.11, p = 0.04), respectively (Table 5).

**Table 4:** Association of polymorphisms with obesity-related traits

Measurements	*FTO* rs9939609	*PPARG* rs1801282	*ADIPOQ* rs4632532	*ADIPOQ* rs182052
	β (CI 95%, P-value)	β (CI 95%, P-value)	β (CI 95%, P-value)	β (CI 95%, P-value)
BMI	**0.46**	-0.004	0.005	-0.003
	**(0.005-0.08, 0.03)**	(−0.05-0.04, 0.87)	(−0.03- 0.04, 0.75)	(−0.04-0.03, 0.84)
Waist circumference (cm)	**0.03**	-0.01	0.004	-0.001
	**(0.006 – 0.07, 0.02)**	(−0.05 – 0.02, 0.51)	(−0.02 – 0.03, 0.76)	(0.03 – 0.02, 0.90)
Arm circumference (cm)	0.03	0.002	0.002	-0.005
	(−0.002 – 0.07, 0.07)	(−0.04 – 0.04, 0.92)	(−0.03 – 0.03, 0.87)	(−0.03 – 0.02, 0.37)
Triceps (mm)	**0.08**	-0.03	-0.02	-0.03
	**(0.007 – 0.16, 0.03)**	(−0.12 – 0.06, 0.53)	(−0.08 – 0.05, 0.59)	(−0.10 – 0.04, 0.75)
Waist/height ratio	**0.03**	-0.01	-0.002	-0.003
	**(0.007 – 0.06, 0.01)**	(−0.04 – 0.02, 0.54)	(−0.02 – 0.02, 0.85)	(−0.03 – 0.02, 0.76)

Linear regression was used to compare obesity measurements by genotype, using additive model adjusted for age and gender. Significant associations (p < 0.05) are indicated in bold. BMI: body mass index.

**Table 5:** Association of polymorphisms with biochemical parameters

Biochemical	*FTO* rs9939609	*PPARG* rs1801282	*ADIPOQ* rs4632532	*ADIPOQ* rs182052
Parameter	β (CI 95%, P-value)	β (CI 95%, P-value)	β (CI 95%, P-value)	β (CI 95%, P-value)
Glucose	0.01	-0.02	0.003	0.001
	(−0.01 – 0.03, 0.36)	(−0.05 – 0.006, 0.11)	(−0.02 – 0.02, 0.75)	(−0.02 – 0.02, 0.95)
Cholesterol	**0.06**	0.01	-0.01	-0.006
	**(001 – 0.11, 0.02)**	(−0.05 – 0.07, 0.72)	(−0.05 – 0.03, 0.64)	(−0.05 – 0.04, 0.77)
Triglycerides	0.07	**-0.11**	0.04	0.04
	(−0.02 – 0.15, 0.14)	**(−0.22 – −0.003, 0.04)**	(−0.04 – 0.11, 0.30)	(−0.04 – 0.12, 0.30)
HDL	-0.02	0.01	**-0.06**	-0.05
	(−0.09 – 0.05, 0.56)	(−0.07 – 0.09, 0.78)	**(−0.12 – −0.007, 0.03)**	(−0.11 – 0.01, 0.12)
LDL	**0.12**	0.04	0.03	0.02
	**(0.03 – 0.22, 0.01)**	(−0.07 – 0.16, 0.47)	(−0.05 – 0.11, 0.43)	(−0.06 – 0.10, 0.63)

Linear regression was used to compare biochemical measurements by genotype, using additive model adjusted for age, gender and body mass index. Significant associations (p < 0.05) are indicated in bold.

## Discussion

In this study, several risk factors of obesity were evaluated in a Mexican children population. The results revealed that genetic factors were associated with obesity related traits, while the obesogenic environment had no effect. The frequency of risk genotypes in *FTO* rs9939609*, PPARG* rs1801282, and *ADIPOQ* rs4632532 and rs182052 was different from what has been reported in other populations. This was expected as a result, since studies show that there is variability in frequencies of polymorphisms in different populations of the same ethnicity (Table 3). In regard to the association of these genes with obesity, most studies report OR's in the range of 1.2 to 2, similar to our results, indicating that these genes are of low penetrance and their contribution to the development of obesity is small. However, the presence of several genetic factors together with other factors could have a significant effect.

The presence of polymorphism *FTO* rs9939609 was associated with an increment in BMI, waist circumference and waist/height ratio. Waist/height ratio measures the abdominal fat in any gender or age and is a better metabolic risk predictor than BMI (Browning *et al.*, 2010; Kodama *et al.*, 2012). This result is in agreement with other studies in different populations (León-Mimila *et al.*, 2013; Luczynski *et al.*, 2012; Villalobos-Comparán *et al.*, 2008; Xi *et al.*, 2010). We also found that the A allele increases the risk of alterations in the lipid profile (cholesterol and HDL levels), as reported by other groups (Villalobos-Comparán *et al.*, 2008; Mascarenhas-Melo *et al.*, 2013). This supports the suggested activity of FTO protein, in conjunction with C/EBP, as a co-activator of PPAR gamma, which is involved in adipocyte functions, such as lipid metabolism and differentiation (Wu *et al.*, 2010).

The polymorphism *PPARG* Pro12Ala (rs1801282) has been studied in different populations with contradictory results; in some studies Ala allele was associated with obesity (Bhatt *et al.*, 2012; Morini *et al.*, 2008), whereas in a Chinese population, it was reported as a protecting factor (Wang *et al.*, 2013). In our study, we did not find an association under any model. However, this polymorphism showed a negative association with triglycerides level (β = −0.11, p = 0.04). This can be supported by *in vitro* evidence, which shows that allele Ala presents less affinity to DNA, and as a result, the ability to induce lipogenesis is decreased (Larsen *et al.*, 2003). The different results reported for *PPARG* rs1801282 may be due to PPARG being a transcription factor that responds to the environment (high fatty acids diet) and shows epistasis with other polymorphisms in the same gene (rs2938392, rs1175542, rs1175544) and other genes (ADB3R). Therefore, specific features of the obesogenic environment may influence associations according to the population analyzed.

*ADIPOQ* rs4632532 showed an association with decreased HDL levels. The polymorphisms evaluated in this study are associated with obesity in Afro-Americans (Bostrom *et al.*, 2008) and with increased BMI, waist circumference, fasting insulin levels and skin fold thickness in Mexicans living in USA (Richardson *et al.*, 2006; Sutton *et al.*, 2005). However, those studies did not find an association with HDL levels. Adiponectin participates in the beta-oxidation of fatty acids (Kadowaki and Yamauchi, 2005), so it is possible that the polymorphic gene impacts the transport of fatty acids (in HDL) into plasma. Accordingly, a positive correlation between HDL and adiponectin levels has been reported elsewhere (Mascarenhas-Melo *et al.*, 2013). Although we did not measure plasma adiponectin, these studies support our results.

In addition to genetic factors, the obesogenic environment including diet, physical activity and screen time was evaluated, but no association was found between environmental factors and obesity or gain in anthropometric measurements. Similar results were found in the National Survey 2006 (Flores *et al.*, 2009). This study presents inaccuracies in diet records of both obese and non-obese groups. Therefore, energy intake could have been wrongly associated with obesity. This imprecision is frequently caused by the use of questionnaires as tools for diet intake assessment. The use of dietary diaries could overcome the problem; however, that method becomes impractical when large populations are surveyed.

The findings of our study are important because there are only two other studies that show association of *FTO* rs9930609 with obesity or obesity-related traits and modified lipid profile in a Mexican population. In addition, the polymorphisms of *PPARG* rs1801282 and *ADIPOQ* rs4632532 were not assessed in those previous studies. However, limitations in our study include a small sample size and difficulty determining the obesogenic environment factors.

In conclusion, this study analyzed environment and genetic aspects of obesity and found significant associations between *PPARG* rs1801282 and triglycerides levels, *ADIPOQ* rs4632532 and HDL levels, and *FTO* rs9939609 with cholesterol, LDL and anthropometric measurements.

## References

[B1] Bhatt S P, Misra A, Sharma M, Luthra K, Guleria R, Pandey RM, Vikram N K (2012). Ala/Ala genotype of Pro12Ala polymorphism in the peroxisome
proliferator-activated receptor-γ2 gene is associated with obesity and insulin
resistance in Asian Indians. Diabetes Technol Ther.

[B2] Bostrom MA, Freedman BI, Langefeld CD, Liu L, Hicks PJ, Bowden DW (2008). Association of adiponectin gene polymorphisms with type 2 diabetes in
an African American population enriched for nephropathy. Diabetes.

[B3] Browning LM, Hsieh SD, Ashwell M (2010). A systematic review of waist-to-height ratio as a screening tool for
the prediction of cardiovascular disease and diabetes: 05 could be a suitable
global boundary value. Nutri Res Rev.

[B4] Casanueva E, Kaufer MPAB (2008). Nutriología Médica.

[B5] Cole TJ, Bellizzi MC, Flegal KM, Dietz WH (2000). Establishing a standard definition for child overweight and obesity
worldwide: International survey. BMJ.

[B6] De Onis M, Onyango AW, Borghi E, Siyam A, Nishida C, Siekmann J (2007). Development of a WHO growth reference for school children and
adolescents. Bull WHO.

[B7] Filippi E, Sentinelli F, Trischitta V, Romeo S, Arca M, Leonetti F, Baroni M G (2004). Association of the human adiponectin gene and insulin
resistance. Eur J Hum Genet.

[B8] Flores M, Macías N, Rivera M, Barquera S, Hernández L, García-Guerra A, Rivera JA (2009). Energy and nutrient intake among Mexican school-aged children, Mexican
National Health and Nutrition Survey 2006. Salud Púb México.

[B9] Frayling TM, Timpson NJ, Weedon MN, Freathy RM, Lindgren CM, Perry JRB, McCarthy MI (2009). A common variant in the FTO gene is associated with body mass index
and predisposes to childhood and adult obesity. Science.

[B10] Ghoussaini M, Meyre D, Lobbens S, Charpentier G, Clément K, Charles M-A, Froguel P (2005). Implication of the Pro12Ala polymorphism of the PPAR-gamma 2 gene in
type 2 diabetes and obesity in the French population. BMC Med Genet.

[B11] González-Sánchez JL, Serrano Ríos M, Fernández Perez C, Laakso M, Martínez Larrad MT (2002). Effect of the Pro12Ala polymorphism of the peroxisome
proliferator-activated receptor gamma-2 gene on adiposity, insulin sensitivity and
lipid profile in the Spanish population. Eur J Endocrinol.

[B12] He W (2009). PPARγ2 polymorphism and human health. PPAR Res.

[B13] Kadowaki T, Yamauchi T (2005). Adiponectin and adiponectin receptors. Endocr Rev.

[B14] Kodama S, Horikawa C, Fujihara K, Heianza Y, Hirasawa R, Yachi Y, Sone H (2012). Comparisons of the strength of associations with future type 2
diabetes risk among anthropometric obesity indicators, including waist-to-height
ratio: A meta-analysis. Am J Epidemiol.

[B15] Lahiri DK, Nurnberger JI (1991). A rapid non-enzymatic method for the preparation of HMW DNA from blood
for RFLP studies. Nucleic Acids Res.

[B16] Larsen TM, Toubro S, Astrup A (2003). PPARgamma agonists in the treatment of type II diabetes: Is increased
fatness commensurate with long-term efficacy?. Int J Obes Relat Metab Disord.

[B17] León-Mimila P, Villamil-Ramírez H, Villalobos-Comparán M, Villarreal-Molina T, Romero-Hidalgo S, López-Contreras B, Gutiérrez-Vidal R, Vega-Badillo J, Jacobo-Albavera L, Posadas-Romeros C (2013). Contribution of common genetic variants to obesity and obesity-related
traits in Mexican children and adults. PLoS One.

[B18] Luczynski W, Zalewski G, Bossowski A (2012). The association of the FTO rs9939609 polymorphism with obesity and
metabolic risk factors for cardiovascular diseases in Polish
children. J Physiol Pharmacol.

[B19] Mascarenhas-Melo F, Sereno J, Teixeira-Lemos E, Marado D, Palavra F, Pinto R, Rocha-Pereira P, Teixeira F, Reis F (2013). Implication of low HDL-c levels in patients with average LDL-c levels:
A focus on oxidized LDL, large HDL subpopulation, and adiponectin. Mediators Inflamm.

[B20] Morales-Ruán MDC, Hernández-Prado B, Gómez-Acosta LM, Shamah-Levy T, Cuevas-Nasu L (2009). Obesity, overweight, screen time and physical activity in Mexican
adolescents. Salud Púb México.

[B21] Morini E, Tassi V, Capponi D, Ludovico O, Dallapiccola B, Trischitta V, Prudente S (2008). Interaction between PPARgamma2 variants and gender on the modulation
of body weight. Obesity.

[B22] Ong KL, Li M, Tso AWK, Xu A, Cherny SS, Sham PC, Lam KSL (2010). Association of genetic variants in the adiponectin gene with
adiponectin level and hypertension in Hong Kong Chinese. Eur J Endocrinol.

[B23] Richardson DK, Schneider J, Fourcaudot MJ, Rodriguez LM, Arya R, Dyer TD, Almasy L, Blangero J, Stern MP, Defronzo RA (2006). Association between variants in the genes for adiponectin and its
receptors with insulin resistance syndrome (IRS)-related phenotypes in Mexican
Americans. Diabetologia.

[B24] Ridley K, Ainsworth BE, Olds TS (2008). Development of a compendium of energy expenditures for
youth. Int J Behav Nutr Phys Act.

[B25] Rivera-Dommarco J, Shamah-Levy T, Villalpando-Hernández S, González de Cossío T, Hernández Prado B SJ (2001). Encuesta Nacional de Nutrición 1999. Estado Nutricio de niños y mujeres de México.

[B26] Rivero-Vázquez S (2013). Encuesta Nacional de Salud y Nutrición2012. Durango.

[B27] Rodríguez-Ramírez S, Mundo-Rosas V, Jiménez-Aguilar A, Shamah-Levy T (2009). Methodology for the analysis of dietary data from the Mexican National
Health and Nutrition Survey 2006. Salud Públ México.

[B28] Shamah-Levy T, Villalpando-Hernández SRDJ (2007). Resultados de Nutrición de la ENSANUT 2006.

[B29] Silva-Zolezzi I, Hidalgo-Miranda A, Estrada-Gil J, Fernandez-Lopez JC, Uribe-Figueroa L, Contreras A, Jimenez-Sanchez G (2009). Analysis of genomic diversity in Mexican Mestizo populations to
develop genomic medicine in Mexico. Proc Natl Acad Sci U S A.

[B30] Speakman JR, Rance KA, Johnstone AM (2008). Polymorphisms of the FTO gene are associated with variation in energy
intake, but not energy expenditure. Obesity.

[B31] Sutton BS, Weinert S, Langefeld CD, Williams AH, Campbell JK, Saad MF, Bowden DW (2005). Genetic analysis of adiponectin and obesity in Hispanic families: The
IRAS Family Study. Hum Genet.

[B32] Tanofsky-Kraff M, Han JC, Anandalingam K, Shomaker LB, Columbo KM, Wolkoff LE, Yanovski JA (2009). The FTO gene rs9939609 obesity-risk allele and loss of control
over. Am J Clin Nutr.

[B33] Tung Y-CL, Ayuso E, Shan X, Bosch F, O’Rahilly S, Coll AP, Yeo GSH (2010). Hypothalamic-specific manipulation of FTO, the ortholog of the human
obesity gene FTO, affects food intake in rats. PloS One.

[B34] Villalobos-Comparán M, Teresa Flores-Dorantes M, Teresa Villarreal-Molina M, Rodríguez-Cruz M, García-Ulloa AC, Robles L, Huertas-Vázquez A, Saucedo-Villarreal N, López-Alarcón M, Sánchez-Muñoz F (2008). The FTO gene is associated with adulthood obesity in the Mexican
population. Obesity.

[B35] Wang X, Liu J, Ouyang Y, Fang M, Gao H, Liu L (2013). The association between the Pro12Ala variant in the PPARγ2 gene and
type 2 diabetes mellitus and obesity in a Chinese population. PLoS One.

[B36] Wu Q, Saunders R, Szkudlarek-Mikho M, La Serna ID, Chin K-V (2010). The obesity-associated Fto gene is a transcriptional
coactivator. Biochem Biophys Res Commun.

[B37] Xi B, Shen Y, Zhang M, Liu X, Zhao X, Wu L, Wang X (2010). The common rs9939609 variant of the fat mass and obesity-associated
gene is associated with obesity risk in children and adolescents of Beijing,
China. BMC Med Genet.

[B38] Zimmet P, Alberti G, Kaufman F, Tajima N, Silink M, Arslanian S, Caprio S (2007). The metabolic syndrome in children and adolescents-an IDF consensus
report. Diabetes Voice.

